# Palliative care and catastrophic costs in Malawi after a diagnosis of advanced cancer: a prospective cohort study

**DOI:** 10.1016/S2214-109X(21)00408-3

**Published:** 2021-10-29

**Authors:** Maya Jane Bates, Miriam R P Gordon, Stephen B Gordon, Ewan M Tomeny, Adamson S Muula, Helena Davies, Claire Morris, Gerald Manthalu, Eve Namisango, Leo Masamba, Marc Y R Henrion, Peter MacPherson, S Bertel Squire, Louis W Niessen

**Affiliations:** a Department of Family Medicine, Kamuzu University of Health Sciences, Blantyre, Malawi; b Department of Public Health, Kamuzu University of Health Sciences, Blantyre, Malawi; c Department of Clinical Sciences, Liverpool School of Tropical Medicine, Liverpool, UK; d Department of Economics, Global Development Institute, University of Manchester, Manchester, UK; e Malawi Liverpool Wellcome Trust, Clinical Research Programme, Blantyre, Malawi; f Worldwide Hospice Palliative Care Alliance, London, UK; g Department of Planning, Ministry of Health, Lilongwe, Malawi; h Department of Medicine, Queen Elizabeth Central Hospital, Ministry of Health, Blantyre, Malawi; i African Palliative Care Association, Kampala, Uganda; j London School of Hygiene & Tropical Medicine, London, UK; k Johns Hopkins School of Public Health, Baltimore, MD, USA

## Abstract

**Background:**

Inclusive universal health coverage requires access to quality health care without financial barriers. Receipt of palliative care after advanced cancer diagnosis might reduce household poverty, but evidence from low-income and middle-income settings is sparse.

**Methods:**

In this prospective study, the primary objective was to investigate total household costs of cancer-related health care after a diagnosis of advanced cancer, with and without the receipt of palliative care. Households comprising patients and their unpaid family caregiver were recruited into a cohort study at Queen Elizabeth Central Hospital in Malawi, between Jan 16 and July 31, 2019. Costs of cancer-related health-care use (including palliative care) and health-related quality-of-life were recorded over 6 months. Regression analysis explored associations between receipt of palliative care and total household costs on health care as a proportion of household income. Catastrophic costs, defined as 20% or more of total household income, sale of assets and loans taken out (dissaving), and their association with palliative care were computed.

**Findings:**

We recruited 150 households. At 6 months, data from 89 (59%) of 150 households were available, comprising 89 patients (median age 50 years, 79% female) and 64 caregivers (median age 40 years, 73% female). Patients in 55 (37%) of the 150 households died and six (4%) were lost to follow-up. 19 (21%) of 89 households received palliative care. Catastrophic costs were experienced by nine (47%) of 19 households who received palliative care versus 48 (69%) of 70 households who did not (relative risk 0·69, 95% CI 0·42 to 1·14, p=0·109). Palliative care was associated with substantially reduced dissaving (median US$11, IQR 0 to 30 *vs* $34, 14 to 75; p=0·005). The mean difference in total household costs on cancer-related health care with receipt of palliative care was −36% (95% CI −94 to 594; p=0·707).

**Interpretation:**

Vulnerable households in low-income countries are subject to catastrophic health-related costs following a diagnosis of advanced cancer. Palliative care might result in reduced dissaving in these households. Further consideration of the economic benefits of palliative care is justified.

**Funding:**

Wellcome Trust; National Institute for Health Research; and EMMS International.

## Introduction

Globally, 18 million new cases of cancer were recorded in 2018 and 9·5 million people with cancer died.^1^ By 2030, a 70% increase in annual cancer cases and deaths is predicted in Africa.^2^ Cancer diagnosis has profound consequences for households in low-income and middle-income countries. A recent study of more than 9000 patients with cancer in southeast Asia reported that 75% of patients had either died or faced financial catastrophe 12 months from diagnosis.^3^ Studies from some comparative settings in Uganda and South Africa report high mortality, catastrophic social and financial consequences, as well as accompanying psychological (anxiety) and spiritual (transcendent) morbidity.^4^ For the few people who access potentially curative therapy, loss to follow-up rates reported in Malawi are high.^5^

Palliative care is part of the continuum of care needed to tackle the heavy burden of serious health-related suffering experienced by patients with chronic non-communicable diseases, although it is widely unavailable and has not yet been the subject of extensive research in low-income settings in countries in Africa.^6^, ^7^ Positive health and economic benefits of palliative care have been reported in systematic reviews, although there are inherent challenges in estimating cost-effectiveness in populations with life-limiting illness.^8^, ^9^ Much of the current literature is from high-income settings and describes the effect of palliative care on cost savings at a health system level. Current approaches have largely failed to capture relevant data from low-income and middle-income countries, where cost savings at household level are acutely important for patients and families, and to inform policy makers.^10^, ^11^

A study in South Africa reported that palliative care reduced repeat admissions to hospital.^12^ Patients and families affected by advanced cancer in Malawi valued palliative care for pain and symptom control, facilitating reintegration into society, enabling patients to return to household livelihood activities or paid work, which benefitted wellbeing.^13^ Timely and compassionate advice for families could reduce or stop the urge to find a cure by so-called doctor shopping.^14^ Hitherto, there are no data from countries in Africa reporting household level costs of health care in settings of advanced cancer, and the potential for palliative care to prevent or reduce financial catastrophe.

**Research in context**

**Evidence before this study**

We searched MEDLINE/PubMed and CINAHL/EBSCO, Web of Science, EconLit, Ovid MEDLINE, Global Health, and African Journals Online to identify studies reporting on household costing in low-middle-income and middle-income countries relevant to a palliative care patient population. Reference lists from identified studies were searched to identify further literature. Searches were limited to articles published between Jan 1, 1992 and Dec 31, 2020 and the terms used were: Palliative care: MH (“Palliative Care”) OR MH (“Palliative Medicine”) OR end of life” OR “terminal illness*” OR “terminal care” OR “end-of-life”; Cost of illness: MH (“Cost of Illness”) “economic burden” OR “household burden” OR “out of pocket” OR “out-of-pocket” OR “financial burden” OR “financial strain” OR “cost of care” OR “cost of illness” OR “direct cost*” OR “indirect cost*” OR “illness cost*” OR “financial stress” OR “catastrophic expenditure” OR “poverty reduction”; Context: LMIC MH (“Developing Countries”) ”developing countr*” OR LMIC OR “low resource” OR “low income” OR “low to middle income” OR “low-to-middle income” OR “least developed countr*” OR “underdeveloped countr*” OR “poor countr*” OR “underdeveloped nation*” OR “least developed nation*” OR “low and middle-income countries”. 30 articles were identified. Studies report high levels of catastrophic expenditure following a diagnosis of cancer in low-income and middle-income countries. Palliative care was mentioned in four papers (India, China, Colombia, global) but no published studies explored the impact of palliative care on household expenditure on health care or catastrophic expenditure. Two papers known to the authors explored the policy relevance of this topic, and one paper reported poverty reduction in a retrospective study conducted among households receiving palliative care in rural India.

**Added value of this study**

Levels of catastrophic expenditure are high after a diagnosis of advanced cancer in Malawi. Observed differences in total household expenditure and catastrophic costs for households receiving palliative care did not reach statistical significance due to limited access to palliative care in the study population.

**Implications of all the available evidence**

Palliative care might contribute to poverty reduction at household level in LMIC through reducing additional burdens of household expenditure on health care in the context of advanced disease. Larger studies with improved access to palliative care are needed to determine whether catastrophic household expenditure is reduced through receipt of palliative care, in the context of cancer or other advanced conditions.

By use of a societal perspective, we aimed to investigate whether total household costs on health care are associated with receipt of palliative care in Malawi after a diagnosis of advanced cancer.

## Methods

### Study design

We undertook a prospective observational study among households in which a patient had received a new diagnosis (between January and July 2019) of advanced cancer at Queen Elizabeth Central Hospital, Blantyre, Malawi between Jan 16 and July 31, 2019. Given the relatively limited survival times anticipated for patients affected by advanced cancer, data were gathered on health-care costs related to cancer illness as well as health-related quality of life (HRQoL) between diagnosis and 6 months following diagnosis.

Queen Elizabeth Central Hospital is one of four tertiary referral teaching hospitals in Malawi. Palliative care services at the hospital fulfil criteria for African Palliative Care Association Level 3—ie, specialist services, including availability of morphine at site and in the home, and degree-level training represented in the team.^15^ At the time of the study, adult palliative care services in this hospital comprised two clinicians (one doctor and one clinical officer), four nurses, a part time chaplain or driver, and a cleaner. New patients were assessed using a form to identify physical, psychological, social, and spiritual needs and concerns of patients and caregivers. Outpatient and home visiting services took place. Referrals to palliative care were made at the discretion of attending clinicians from wards and outpatient clinics. Common reasons for referral included pain and symptom relief, counselling of patients and families on disease understanding, and social needs such as nutritional support. Palliative care services were delivered concurrently with other specialist services; palliative care could be started (or stopped) at any stage of the patient's illness journey as was appropriate to their needs.

Basic costs of health services at the hospital, including oncology and palliative care, were met by government funding through the Ministry of Health. Following registration at minimal cost, services were provided free of charge to the patient. Supplementary donor funding for palliative care supports some salaries of health workers, medications, nutritional support, and transportation for home visits.

This study has undergone ethical review by, and received approval from, the College of Medicine Research Ethics Committee in Blantyre, Malawi, and the Liverpool School of Tropical Medicine Research Ethics Committee. The study protocol has been published elsewhere.^16^

### Participants

Participant households (patient–carer dyads) were recruited sequentially from specialist clinics at the hospital when a patient was identified with a new diagnosis of advanced cancer (any one of Kaposi's sarcoma, cervical cancer, oesophageal cancer, or hepatocellular carcinoma; appendix p 4). Patients were invited to take part in the study if 18 years or older, living less than 50 km from the hospital, with stable comorbidities (ie, temperature <37·4°C, blood pressure ≤140/90 mm Hg, WHO performance score ≤2), and an estimated prognosis of at least 3 months as determined by an experienced palliative care clinician. Following study enrolment, patients were asked to identify an unpaid family caregiver from the same household. Health-care use and related household costs, including sale of assets and loans taken out (dissaving), were recorded. HRQoL was recorded using the Chichewa version of the EuroQoL EQ-5D-3L tool.^17^ All costs for health care for cancer-related illness were collated between diagnosis and 6 months after diagnosis using the Patient and Carer Cancer Cost Survey.^18^ Repeat visits at 1 month and 3 months were made at a site of preference chosen by the respondent (ie, home, nearby health centre, or hospital). The frequency of visits was designed to reduce recall time to improve the accuracy of self-reported costs.^19^ If a follow-up appointment was missed, patients were asked in the subsequent interviews about all costs and dissaving since their last attended appointment. We recorded receipt of palliative care as a binary exposure (yes/no) by household self-report and verified by manual checking of clinical records and hospital data management reports.

Households were defined as rural if they were located outside Blantyre urban administrative boundaries. Self-reported poverty levels were assessed based on World Bank definitions.^20^ In addition, households reported asset ownership and were divided into poverty tertiles derived from scores calculated from a locally developed proxy means test for poverty (appendix pp 11–13). Tertiles were used due to the relatively small sample size.

### Statistical analysis

Comparing single variables (a response variable was the total household costs of health care from diagnosis to 6 months as a proportion of household income, predictor was the receipt of palliative care) a sample size of 55 households was required to detect a medium effect size at 6 months (as determined by Cohen's f^2^=0·15). All power analyses were based on α=0·05; power=0·8, using two-sided tests. Accounting for an estimated 50% exclusions and 20% dropout, the cohort needed a sample size of 138 households. The study sample was partly based on feasibility, with an estimated 225 patients available for recruitment over a 6 month period.

Analysis was completed using StataCorp version 15, R version 4.1.0 and Microsoft Excel (Windows 10). STROBE and CHEERS guidelines were used for reporting.

### Procedures and outcomes

Only households reporting data for the primary outcome (total household costs on health care from diagnosis to 6 months after diagnosis) were included in the descriptive analysis. Continuous variables were summarised as means (with 95% CI), and medians (with IQR) if data were skewed. Frequency tables and percentages were used for categorical variables. Wilcoxon rank sum test and Spearman correlation were used for comparisons. Fisher's exact test and relative risk calculations were used to explore catastrophic costs on health care, comparing households that received palliative care with those that did not.

We formulated multiple linear regression models to investigate associations between palliative care receipt and total household costs on health over the 6 months following diagnosis, expressed as a proportion of household income. We derived annual household income from monthly household income before the onset of symptoms. Total household costs included all direct medical and non-medical out-of-pocket expenditure, as well as indirect costs through lost productivity time for both patients and carers. Models were constructed based on previous literature, and controlled for socioeconomic status, health seeking behaviour, health status of patient at diagnosis, and cancer type (appendix pp 2–3).

Dissaving was defined as the sale of assets (eg, bicycles or land) or the acquisition of loans. Dissaving is regarded as a coping strategy to access health services in low-income settings.^21^ It might be more accurately recalled by household members than precise retrospective details of health-care related out-of-pocket expenditure and has been considered as a potential proxy marker of catastrophic costs.^22^ Households were deemed to have faced catastrophic costs on health care if their total costs were greater or equal to 20% of their annual income before illness onset.^23^ Catastrophic costs were described as relative risks by category, comparing those who received palliative care with those who did not.

Median EuroQoL EQ-5D-3L utility scores (Zimbabwe tariff) and visual analogue scale (VAS) scores were recorded at each timepoint. The two-sample Wilcoxon rank test was used for HRQoL comparisons between those who had received palliative care and those who had not, and Spearman correlation tests were used to investigate the relationship between VAS scores and utility scores. The unadjusted hazard of death was estimated using the Kaplan-Meier survival estimator, with survival disaggregated by poverty status, receipt of palliative care, and cancer type (appendix pp 7–9). No multiple testing corrections were applied.

After their final study visit, as per local ethical requirements at the time of the study, households received local currency equivalent of US$10 compensation per visit. Data were entered anonymously on Open Data Kit software using locked hand-held devices for data collection. These were stored in a locked room when not in use. Data were uploaded at the end of each working day onto a password-protected laptop and uploaded onto a secure server at the Malawi Liverpool Wellcome Trust Clinical Research Programme (Blantyre, Malawi). Data were erased once uploaded to a secondary server. The database was backed up on a locked and encrypted hard drive.

### Role of the funding source

The funder of the study had no role in study design, data collection, data analysis, data interpretation, or writing of the report. The corresponding author had full access to all the data in the study and had final responsibility for the decision to submit for publication.

## Results

Between Jan 16 and July 31**,** 2019, 156 households, comprising 280 individuals (156 patients and 124 caregivers), in which a patient had been newly diagnosed with cancer were assessed for eligibility, of whom 150 households comprising 271 individuals (150 patients and 121 caregivers) were recruited (table 1). At 6 months, 89 (59%) households representing 153 individuals (89 patients and 64 caregivers) were available for analysis, 19 (21%) had received palliative care and 70 (79%) had not. Between diagnosis and 6 months, 55 (37%) of 150 patients had died, with 22 (40%) of these deaths having occurred before first follow-up at 1 month (appendix p 6). Median time from recruitment to death was 53 days (appendix pp 7–9). A total of five (3%) of 150 households missed one interview (two at 1 month and three at 3 months) but returned for the following interview. Six (4%) of 150 households were lost to follow-up (figure, appendix p 6).

**Table 1:** Household, patient, and carer demographics at 6 months following a diagnosis of advanced cancer, by receipt of palliative care

	**Did not receive palliative care (n=70)**	**Received palliative care (n=19)**
**Household**		
Rural	42 (60%)	6 (32%)
Urban	28 (40%)	13 (68%)
Most poor	23 (33%)	6 (32%)
Poor	23 (33%)	5 (26%)
Least poor	24 (34%)	8 (42%)
Living in extreme poverty	51 (73%)	9 (47%)
**Patient**		
Female	56 (80%)	14 (74%)
Male	14 (20%)	5 (26%)
18–40 years	16 (23%)	4 (21%)
41–60 years	36 (51%)	11 (58%)
>60 years	18 (26%)	4 (21%)
Cervical cancer	50 (71%)	10 (53%)
Oesophageal cancer	16 (23%)	3 (16%)
Kaposi's sarcoma	3 (4%)	5 (26%)
Liver cancer	1 (1%)	1 (5%)
Married	44 (63%)	8 (42%)
Single or divorced	7 (10%)	7 (37%)
Widowed	19 (27%)	4 (21%)
**Carer**		
Female	34/49 (69%)	13/15 (87%)
Male	15/49 (31%)	2/15 (13%)
18–40 years	26/49 (53%)	6/15 (40%)
41–59 years	17/49 (35%)	8/15 (53%)
60–89 years	6/49 (12%)	1/15 (7%)

Data are n/N (%), unless otherwise stated.

**Figure:**
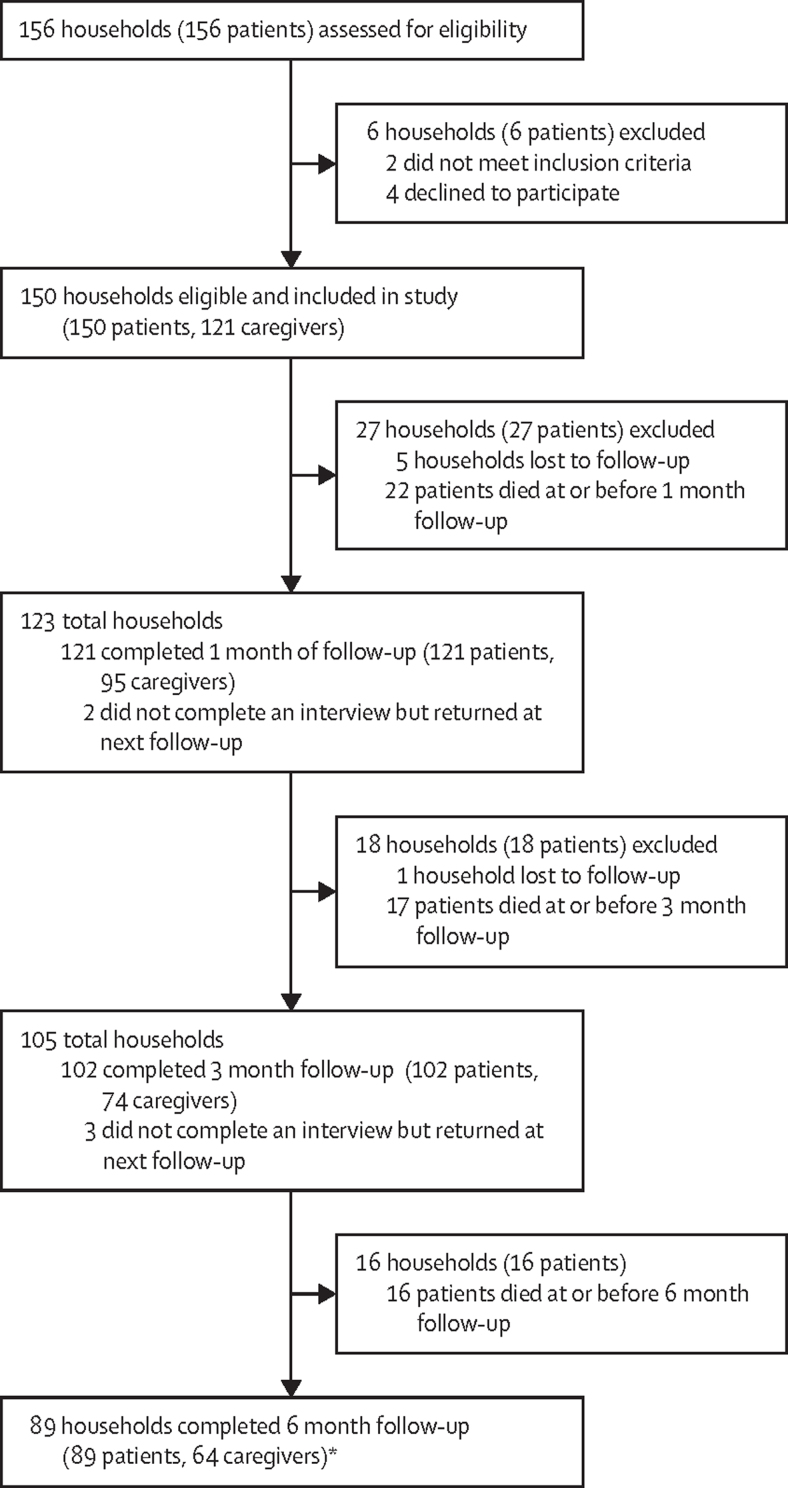
Trial profile *19 households received palliative care, and 70 households did not receive palliative care.

Median annual household income before illness onset was $204 (IQR 84–660), 60 (67%) of 89 households were living in extreme poverty (≤$1·90 per day). 48 (54%) of 89 households were rural. The median age of patients was 50 years (IQR 40–57), and for caregivers was 40 years (32–49). 70 (79%) patients and 47 (73%) caregivers were female. Of the 89 patients, 60 (67%) had cervical cancer, 19 (21%) had oesophageal cancer, eight (9%) had Kaposi's sarcoma, and two (2%) had hepatocellular carcinoma (table 1).

Median annual household income before illness onset for those who received palliative care was $537 (IQR 107–821) and for those who did not receive palliative care was $179 (82–537; p=0·135). In the 6 months after diagnosis, median total household costs on health were $50 (IQR 11–101) for those who received palliative care and $55 (28–91) for those who did not (p=0·704; table 2). Median direct costs were $6 (IQR 4–26) for those receiving palliative care and $12 (0–21) for those who did not receive palliative care (p=0·252); median indirect costs were $36 (IQR 5–56) versus $33 (13–56; p=0·980).

**Table 2:** Household income, health-related costs at 6 months, and dissaving by receipt of palliative care

	**Did not receive palliative care**	**Received palliative care**
Household costs on health	$55 (28–91)	$50 (11–101)
Household income before illness	$179 (82–537)	$537 (107–821)
Household costs as proportion of household income	0·278 (0·085–0·692)	0·086 (0·037–0·579)
Dissaving at 6 months	$34 (14–75)	$11 (0–30)

Data are median US$,2019, (IQR), unless otherwise specified.

Expressed as a proportion of household income, total household cost on health for those who received palliative care was 0·086 (IQR 0·037–0·579) compared with 0·278 (0·085–0·692) for those who did not receive palliative care (p=0·126). After adjustment for other relevant variables through multiple linear regression analysis, the mean difference in total household costs on cancer related health care as a proportion of household income was −36% (95% CI −94 to 594) for those who received palliative care compared with those who did not receive palliative care (p=0·707; table 3). Two sensitivity analyses were done, over a shorter 3 month period following diagnosis, and using a different method of calculating the indirect costs. At 3 months, mean difference in total household costs on health care were −54% (95% CI −95 to 351, p=0·533) with receipt of palliative care. Using minimum wage for calculating indirect costs at 6 months this difference was −46% (95% CI −95 to 490, p=0·608; table 3; appendix p 5).

**Table 3:** Unadjusted and adjusted percentage difference in total household costs of health care as a proportion of household income following a diagnosis of advanced cancer, by receipt of palliative care

	Unadjusted difference*(95% CI)	Adjusted difference*(95% CI)
**Household**		
Received palliative care	−62·7% (−97·0 to 309·0)	−36·1% (−94·1 to 594·0)
Urban	−4·6% (−86·0 to 655·0)	155·0% (−65·1 to 1770·0)
Most poor	103·0% (−75·0 to 1550·0)	..†
Poor	−22·7% (−91·0 to 544·0)	..†
Least poor	−35·3% (−92·0 to 403·0)	..†
Living in extreme poverty	4180·0% (513·0 to 29800·0)	..†
**Patient**		
Female	709·0% (−24·0 to 8480·0)	−44·5% (−95·9 to −4980·0)
Married	18·6% (−84·0 to 775·0)	−16·7% (−89·7 to 575·0)

* Coefficients have been back transformed from the log–linear regressions and so can be interpreted as the % change in the health share of income with a one-unit change in the independent variable.

† No data where the explanatory variable was not included in the regression model.

57 (64%) of 89 households experienced catastrophic costs (table 4). Nine (47%) of 19 households who received palliative care experienced catastrophic costs compared with 48 (69%) of 70 who did not receive palliative care (relative risk 0·69, 95% CI 0·36–1·04, p=0·109). Catastrophic costs were more commonly experienced by rural households (37 [77%] of 48) than by urban households (20 [49%] of 41; p=0·008). Median dissaving at 6 months was $11 (IQR 0–30) for those receiving palliative care and $34 (14–75) for those who did not (p=0·005).

**Table 4:** Catastrophic health-related costs (20% threshold of household income) following a diagnosis of advanced cancer, by receipt of palliative care (n=89)

	**No palliative care**	**Palliative care**	**Risk ratio (95% CI)**
**Household**			
All	48/70 (69%)	9/19 (47%)	0·69 (0·36–1·04)*
Rural	35/42 (83%)	2/6 (33%)	0·40 (0·00–0·88)†
Most poor	17/23 (74%)	2/6 (33%)	0·45 (0·00–1·05)†
Poor	14/23 (61%)	3/5 (60%)	0·99 (0·29–1·92)†
Least poor	17/24 (71%)	4/8 (50%)	0·71 (0·19–1·29)†
Extreme poverty	41/51 (80%)	7/9 (78%)	0·97 (0·60–1·141)†
**Patient**			
Male	8/14 (57%)	1/5 (20%)	0·35 (0·06–2·14)†
Female	40/56 (71%)	8/14 (57%)	0·80 (0·49–1·29)†
Kaposi's sarcoma	2/3 (66%)	0/5 (0%)	0·00 (NA)
Cervical cancer	38/50 (76%)	7/10 (70%)	0·92 (0·51–1·342)†
Oesophageal cancer	8/16 (50%)	1/3 (33%)	0·67 (0·00–2·13)†
Hepatocellular carcinoma	0/1 (0%)	1/1 (100%)	NA (NA)
**Other**			
Less than median household income before illness	33/37 (89%)	6/7 (86%)	0·96 (0·62–1·23)†
Greater than median total household costs on health	32/36 (89%)	6/8 (75%)	0·84 (0·45–1·17)†

Data are n/N (%), unless otherwise stated. NA=not available.

* Confidence interval obtained using a small–sample–adjusted unrestricted maximum likelihood estimator and the Wald normal approximation.

† Confidence interval obtained by using the percentile method and bootstrapping (drawing repeated binomial samples for each group with the empirical probability of experiencing catastrophic costs).

Health-related quality-of-life scores expressed in mean utility scores at diagnosis were 0·668 (95% CI 0·628–0·707) for patients and 0·826 (0·799–0·853) for carers, and 6 months after diagnosis were 0·590 (0·534–0·646) for patients and 0·831 (0·799–0·863) for carers. Mean VAS scores were 39 (95% CI 35–42) for patients and 32 (29–35) for carers at diagnosis and 23 (20–25) for patients and 18 (15–22) for carers (appendix p 10). Comparison of utility and VAS scores 6 months after diagnosis showed no difference between people who received palliative care versus those who did not in patients (mean utility scores of 0·537 [95% CI 0·412–0·662] *vs* 0·606 [0·543–0·670], p=0·150; VAS scores of 23 [18–28] *vs* 22 [20–25], p=0·616) or caregivers (mean utility scores of 0·842 [0·768–0·917] *vs* 0·828 [0·792–0·864], p=0·647, VAS scores of 22 [6–38] *vs* 17 [15–19], p=0·284; table 5; appendix p 10). Patient HRQoL utility scores were negatively correlated with VAS scores at 6 months (data not presented). There was no correlation between total costs on health care at 6 months and HRQoL, irrespective of receipt of palliative care (data not presented).

**Table 5:** Patient and carer HRQoL utility and VAS scores from diagnosis to 6 months after diagnosis of advanced cancer by receipt of palliative care (n=89)

	**HRQoL health index (maximum=1)**			**VAS score (maximum=100)**		
	All (95% CI)	Not received palliative care (95% CI)	Received palliative care (95% CI)	All (95% CI)	Not received palliative care (95% CI)	Received palliative care (95% CI)
**Patients**						
Diagnosis	0·668 (0·628–0·707)	0·668 (0·625–0·711)	0·666 (0·564–0·767)	39 (35–42)	38 (34–42)	42 (33–51)
1 month	0·609 (0·556–0·662)	0·641 (0·589–0·692)	0·461 (0·284–0·639)	35 (32–39)	35 (31–39)	37 (25–48)
3 months	0·647 (0·603–0·691)	0·652 (0·602–0·702)	0·627 (0·523–0·732)	31 (27–34)	30 (26–33)	35 (25–44)
6 months	0·590 (0·534–0·646)	0·606 (0·543–0·670)	0·537 (0·412–0·662)	23 (20–25)	22 (20–25)	23 (18–28)
**Carers**						
Diagnosis	0·826 (0·799–0·853)	0·827 (0·793–0·860)	0·824 (0·776–0·871)	32 (29–35)	32 (29–36)	33 (25–42)
1 month	0·827 (0·797–0·858)	0·812 (0·777–0·847)	0·869 (0·804–0·933)	27 (23–31)	28 (24–33)	24 (17–32)
3 months	0·838 (0·802–0·874)	0·840 (0·796–0·884)	0·831 (0·770–0·891)	25 (22–28)	25 (21–28)	25 (17–33)
6 months	0·831 (0·799–0·863)	0·828 (0·792–0·864)	0·843 (0·768–0·917)	18 (15–22)	17 (15–19)	22 (6–38)

Data are mean (95% CI). HRQoL=health related quality of life. VAS=visual analogue scale.

## Discussion

Advanced cancer diagnosis is associated with catastrophic costs of health care and is a source of household poverty in Malawi. Palliative care might reduce household costs on cancer related illness while maintaining quality of life. Reductions in dissaving were associated with receipt of palliative care in general. This might partly be explained by the higher median income of households receiving palliative care, although this suggestion requires further investigation. After controlling for socioeconomic, demographic, and disease related variables, we did not find a significant association between receipt of palliative care and household costs on health care related to advanced cancer at 6 months. Yet, the observed median difference in household costs was in the hypothesised direction and a similar pattern was observed in the sensitivity analyses. To our knowledge, this is the first time data have been gathered and analysed for households affected by advanced cancer in a low-income country setting.

One in five households affected by advanced cancer under routine care received palliative care, which contributed to the study having limited power to measure a significant difference. In a post-hoc calculation, we recalculated a minimum sample size of 500 households would be required to provide at least 80% power to detect a medium difference (Cohen's d=0·5) between groups. Absolute amounts of dissaving were small across all households. Patients from 59% of households were alive 6 months after a diagnosis of advanced cancer, highlighting the importance of palliative care provision well before the last stages (hours and days) of life to mitigate longer periods of serious health-related suffering.

A key goal of universal health coverage is for individuals and communities to receive the health services they need without suffering financial hardship. This aim encompasses the full spectrum of health services, from health promotion to prevention, treatment, rehabilitation, and palliative care.^24^ Data were deliberately gathered from patient–carer dyads in this study, recognising that financial hardship (and any potential cost savings) relating to serious illness is experienced beyond the individual, and, for a household, even beyond the death of the patient. 67% of households analysed in this study were living in extreme poverty before onset of symptoms. This finding is not surprising, reflecting the fact that 71% of the population of Malawi live in extreme poverty.^25^ This background provides context for the burden of catastrophic costs on health care related to cancer, which contributes further to household vulnerability. The World Bank, UN, and WHO have ambitious targets to reduce extreme poverty, recognising the role catastrophic costs play, stating that no one should be left behind.

There are some limitations to the concept of catastrophic costs, particularly in extremely poor households. Among them, many people who are sick forgo treatment rather than have catastrophic costs. This situation is missed in most studies and debates about universal health coverage.^26^ Additional qualitative work could address questions of whether and how receipt of palliative care influences household costs on health. We were unable to do further subgroup analysis because of the small number of households receiving palliative care. Some details of the timing, number, and type of contacts with palliative care services were available from clinical records; however, more detailed descriptors of support activities would help to describe receipt of palliative care as a continuous variable, which would have contributed to a more robust level of quantitative analyses on exposure.

The Global Atlas of Palliative Care states that only 12% of the 57 million adults and children who need palliative care, to reduce serious health-related suffering, currently receive it.^27^ Before starting the study, we considered that it was unethical to randomly assign households to palliative care, opting to use an observational study design conducted within a routine care setting. All recruited households were affected by advanced cancer and fulfilled criteria to receive palliative care at diagnosis. Although services were offered free of charge within a government funded institution, crucial gaps in access to care were noted. Wealthier households in urban areas within the 50 km operating radius were more likely to access services (table 1). Where integrated outpatient services existed that supported referral to palliative care (eg, the dedicated weekly palliative care Kaposi's sarcoma outpatient clinic), patients were more likely to receive care. Recommendations from WHO and the *Lancet* Commission for integrated models of palliative care early in the cancer care pathway require urgent implementation.^28^, ^29^

We also noted the lower receipt of palliative care among people who live in extreme poverty in rural areas, which is where most Malawians live. Integration of services at all levels with effective linkages between hospital and community-based care would reduce the substantial transport costs for households. Studies detailing costs and outcomes associated with established and innovative models of care—eg, mobile outreach, mentorship, and support of staff in rural health facilities and mHealth^30^ might be appropriate.

It is striking that HRQoL utility scores were not negatively related to levels of total household costs on health care. The main purpose of recording HRQoL was to comment on whether any cost savings attributed to palliative care were gained at the expense of quality of life—ie, whether households were preventing increased spending on health-related costs simply by stopping any form of health care. The finding that there were no significant differences in HRQoL metrics (utility scores and VAS scores) between people who received palliative care and those who did not broadly suggests that cost savings were not gained at the expense of HRQoL.

Utility scores were higher than those observed in an earlier study among inpatients at the same institution.^31^ Observed reductions in HRQoL over time were not unexpected, given that patients had advanced (and advancing) cancer. This study was not designed or powered to explore differences in quality-of-life relating to receipt of palliative care. The relevance and sensitivity of the EuroQoL EQ-5D to adequately capture changes in HRQoL in populations with advanced disease is contested.^32^ Higher utility scores should be reflected by higher VAS scores; our finding of negative correlation between utility scores and VAS scores for patients at 6 months provides some indication of difficulties in using this tool and warrants further exploration. No validation of existing HRQoL measures for populations with palliative care needs in low-income and middle-income countries presents difficulties, particularly as reporting of quality-adjusted life-years, disability-adjusted life-years, and incremental cost-effectiveness ratios determine priorities for national Essential Health Packages.^33^

Our study has strengths and limitations due to its purposely modest, single-centre observational design. Although public health services in Malawi are provided free at the point of care, financed by taxation and donor funding, there are limited facilities for cancer care, for example, no radiotherapy. Health financing environments and stage of development of oncology and palliative care services vary considerably within and by region, affecting generalisability of findings. Self-reported cost data might be subject to recall bias, despite short recall periods. Seasonal variations affect income in Malawi's largely rural, subsistence farming economy and could confound findings. Refusal (6%) and loss to follow-up (4%) rates were low and potential selection bias due to loss to follow-up was limited. Loss through death of patients was high, as expected, and these households were largely excluded in the data collection and analyses. Potential systematic errors resulting from study recruitment patterns (clinically stable, majority female outpatients) and through referral patterns to palliative care must be noted. Our analyses do not reflect costs or HRQoL experiences of patients who were less stable at diagnosis.

This prospective descriptive study provides essential data to explore the impact of receipt of palliative care on household costs of health care following a diagnosis of advanced cancer in a low-income country setting. Larger studies should be conducted across various settings using the tools and concepts outlined in this Article. However, sample size requirements would be reduced where access to palliative care is improved. Future work should explore barriers to, and successes in, models of early integration of palliative care for patients with advanced cancer, defining and describing the intervention exposure (palliative care) in more detail. Ways to comprehensively report cost data in households where patients die should be described. Unfortunately, paucity of funding for both hospital and community services and related research will hamper the development of the evidence base. As cancer research advances in low-income and middle-income countries, there is a crucial need for improved operational tools to gather socioeconomic outcomes alongside disease related outcomes in recognition of the high levels of catastrophic costs described in this and other studies. Resource-stratified, evidence-based global guidelines have been published for palliative care.^34^ Based on our findings, these previous calls for new and integrated models of care for non-communicable diseases and other chronic conditions under universal health coverage should also consider including access to publicly funded palliative care.

**This online publication has been corrected. The corrected version first appeared at thelancet.com/lancetgh on November 16, 2021**

## Data sharing

Data will be shared within the research community through an open access repository. In addition to the study protocol, the statistical analysis plan and informed consent forms are available online.

## Declaration of interests

We declare no competing interests.
