# Correction to *Lancet Glob Health* 2021; 9: e1750–57

**DOI:** 10.1016/S2214-109X(21)00524-6

**Published:** 2021-11-16

*Bates MJ, Gordon MRP, Gordon S, et al. Palliative care and catastrophic costs in Malawi after a diagnosis of advanced cancer: a prospective cohort.* Lancet Global Health *2021; **9:** e1750–57*—In this Article, table 5 has been added into the article. This correction has been made as of Nov 16, 2021.

