# Supplementary material for: Palliative care and catastrophic costs in Malawi after a diagnosis of advanced cancer: a prospective cohort study
Source: Lancet Glob Health. 2021 Oct 29;9(12):e1750–7. doi: 10.1016/S2214-109X(21)00408-3 (PMC8600125; doi:10.1016/S2214-109X(21)00408-3)
Supplement: Supplementary appendix [file mmc1.pdf]

THE LANCET
Global Health

Supplementary appendix

This appendix formed part of the original submission and has been peer reviewed. We post it as supplied by the authors.

Supplement to: Bates MJ, Gordon MRP, Gordon SB, et al. Palliative care and catastrophic costs in Malawi after a diagnosis of advanced cancer: a prospective cohort study. *Lancet Glob Health* 2021; published online Oct 29. http://dx.doi.org/10.1016/S2214-109X(21)00408-3.

Appendix to manuscript "Palliative care and catastrophic costs in Malawi following a diagnosis of advanced cancer - a prospective cohort study"

Authors: Maya Jane Bates[1,2], Miriam R.P. Gordon[3], Stephen B Gordon[2,4,], Ewan M Tomeny[2], Adamson S Muula[1], Helena Davies[5], Claire Morris[5], Gerald Manthalu[6], Eve Namisango[7], Leo Masamba[6] Marc Y. R. Henrion[2,4,], Peter MacPherson[2,4,8], S Bertel Squire[2], Louis W Niessen[2,9]

Affiliations:

1. Kamuzu University of of Health Sciences Blantyre, Malawi
2. Liverpool School of Tropical Medicine, UK
3. University of Manchester, UK
4. Malawi Liverpool Wellcome Trust Clinical Research Programme, Blantyre, Malawi
5. [1]Worldwide Hospice Palliative Care Alliance, London, UK
6. Ministry of Health, Government of Malawi, Lilongwe, Malawi
7. African Palliative Care Organisation, Kampala, Uganda
8. London School of Hygiene and Tropical Medicine, UK
9. Johns Hopkins School of Public Health, Baltimore, USA

Corresponding author:

Dr M Jane Bates
Department of Family Medicine, Kamuzu University of Health Sciences
P/Bag 360
Blantyre, 3,
Malawi
mjanebates@gmail.com, Tel: +265 999 208193

1

Details of costing calculations

In the regression model a log linear transformation of the total household costs on health variable was used. A log linear transformation was used on the dependent variable (total health expenditure) as the data were skewed. The multiple linear regression equation is stated below.

$$ln(Total\ health\ costs\ as\ a\ share\ of\ monthly\ income) = \alpha_0 + \alpha_1.Receipt\ of\ palliative\ care + \mathbf{Socioeconomic}.\alpha_2 + \mathbf{Health\ seeking\ behaviour}.\alpha_3 + \mathbf{Health\ at\ diagnosis}.\alpha_4 + \mathbf{Type\ of\ cancer}.\alpha_5$$

Direct costs included all out of pocket expenditure on healthcare related to cancer : transport, medication, consultations, food while seeking care, food requirements due to disease. Indirect costs were calculated using the human capital approach (hourly wage x total time taken for visits to health care providers), accounting for lost income due to health care seeking related to cancer illness. In the six month sensitivity analysis, the national minimum wage[2] rather than reported wage was used to calculate hourly wage included in the indirect cost calculation. The time taken for medical visits for the carer and patient were multiplied by the hourly wage of caregivers and patients. For the analysis it was assumed that all patients have a caregiver. The hourly wage of the patient was calculated from the inflation adjusted self-reported income before the onset of symptoms, and for the carer is the calculated from the mean personal income of caregivers reported at the time of diagnosis. For those who missed an interview, questions were asked about costs since the last interview, so that cost data were deemed to be complete in subsequent interviews. Where 0 values were reported for household revenue or THE costs incurred a value of

[2] 25,000 Malawi Kwacha/month = USD2019 $34/month, December 24th 2020 https://times.mw/new-minimum-wage-starts-january-1/ (accessed 7th July 2021)

1 was used to enable log values to be calculated. Sensitivity analysis was done using the national minimum wage to derive patient and carer hourly wages.

Explanatory variables of regression models

We list the explanatory varaiables used in the regression models in the figure and the text below

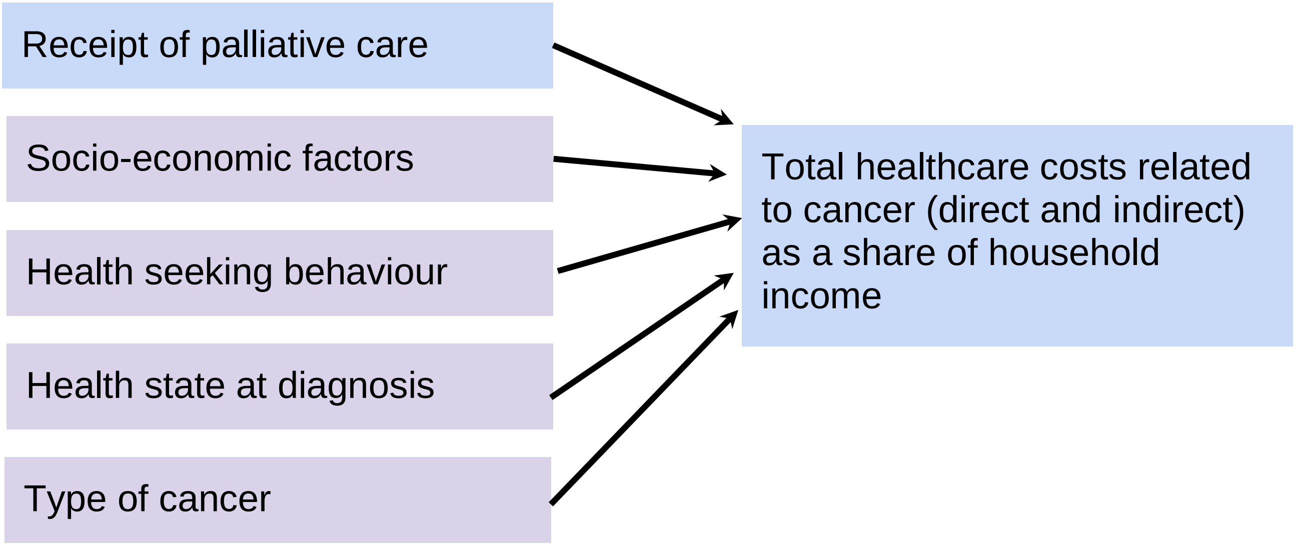

We included receipt of palliative care as a dummy variable and controlled for socioeconomic status, health seeking behaviour, health state of patient at diagnosis, and cancer type as follows:

<u>Socio-economic status</u>: Patient age, sex, marital status, urban or rural dwelling and household size were all included as control variables. Self-reported household annual revenue before the onset of symptoms was adjusted for inflation and included as a control.

<u>Health seeking behaviour</u> : The average direct cost of health care sought before diagnosis per day was divided by self-reported household daily income as a control.

<u>Health state</u> : the length of time from symptom onset to diagnosis and the WHO performance score at diagnosis were included as control variables.

<u>Type of cancer</u>: Differences based on the type of cancer were controlled for.

Diagnostic criteria for advanced cancer

Cancer type	Diagnostic criteria for advanced disease
Kaposi's sarcoma:	ALL patients with a first-time diagnosis on clinical examination by specialist doctor<br>AND<br>assessed as AIDS Clinical Trials Group (ACTG) 'poor risk' category<br>OR<br>ALL with a first-time diagnosis of KS where staging not done
Cervical cancer:	ALL patients with a first-time diagnosis on clinical examination by specialist doctor<br>AND<br>with disease at International Federation of Gynaecology and Obstetrics (FIGO) stage 2 and above<br>OR<br>ALL with a first-time diagnosis (where staging not done)
Oesophageal cancer:	ALL patients with a first-time diagnosis on endoscopy by specialist doctor<br>AND<br>assessed as being inoperable,<br>OR<br>ALL with a first-time diagnosis (where no management plan/staging stated)
Hepatocellular carcinoma	ALL patients identified with a liver mass followed by confirmatory ultrasound with mass >2cm performed by a specialist doctor<br>AND<br>evidence of local mass effect.

Summary of multiple linear regression analysis and sensitivity analyses

Effect of variables on total household costs on health as a proportion of annual household income ((log linear transformed variable), including 95% confidence intervals

	Main regression results (6 months)	Sensitivity Analysis 1 (3 months)	Sensitivity Analysis 2 (6 months using minimum wage)
VARIABLES			
Receipt of palliative care	-36.1%	-54.0%	-45.9%
	(-94.1% - 594%)	(-95.3% - 351%)	(-95% - 490.%)
Age	-0.949%	-1.10%	-0.610%
	(-8.5% - 7.23%)	(-8.16% - 6.49%)	(-8.2% - 7.61%)
Female	44.5%	176.0%	19.5%
	(-95.9% -4980%)	(-90.4% - 7880·0%)	(-96.6% - 4130·0%)
Urban	155%	12.3%	145%
	(-65.1% - 1770%)	(-83% - 642%)	(-66.7% - 1700%)
Married	-16.7%	-77.0%	-11.5%
	(-89.7% - 575%)	(-96.8% - 66.7%)	(-89.1% - 620%)
Household size	52.4%	54.5%	54.2%
	(-3.76% - 141%)	(0.215% - 138%)	(-2.71% - 145%)
Monthly income (before symptoms)	-0.00426%***	-0.00375%***	-0.00461%***
	(-0.00672% - -0.0018%)	(-0.00607% - -0.00144%)	(-0.00708% - -0.00215%)
Daily health spending/daily income (before diagnosis)	0.0721%***	0.250%***	0.0718%***
	(0.0364% - 0.108%)	(0.171% - 0.329%)	(0.036% - 0.108%)
HIV positive	22.6%	17.3%	25.3%
	(-83.6% - 816%)	(-82.2% - 674%)	(-83.3% - 840%)
Days with symptoms	0.164%	0.165%	0.154%
	(-0.0624% - 0.391%)	(-0.0482% - 0.379%)	(-0.073% - 0.382%)
Other variables included		WHO score at diagnosis and type of cancer	
Constant	-86.1%	-62.2%	-87.8%
	(-100% - 8750%)	(-99.9% - 15600%)	(-100% - 7780%)
Observations	89	86	89
R-squared	0.395	0.522	0.423

Demographic summary of households, patients, and carers, all recruited, those surviving to study completion, those who died, by receipt of palliative care

	all recruited		received palliative care		completing follow up		received palliative care		patient died		received palliative care		loss to follow up
	#	%	#	%	#	%	#	%	#	%	#	%	#
households	150		30		89		19		55		10		6
rural	81	*54*	8	*27*	48	*54*	6	*32*	31	*56*	2	*20*	2
urban	69	*46*	22	*73*	41	*46*	13	*68*	24	*44*	8	*80*	4
most poor	50	*33*	7	*23*	29	*33*	6	*32*	21	*38*	1	*10*	
poor	50	*33*	11	*37*	28	*31*	5	*26*	19	*35*	6	*60*	3
least poor	50	*33*	12	*40*	32	*36*	8	*42*	15	*27*	3	*30*	3
	5		5		5		5		4		4	*40*	
>median monthly rev before illness (12,100MK)	74	*49*	18	*60*	44	*49*	12	*63*	26	*47*	6	*60*	4
>median total health costs (37,867MK)					45	*51*	8	*42*	31	*56*	7	*23*	
>median dissaving (20,000MK)					44	*49*	6	*32*	28	*51*	6	*22*	
patients	150		30		89		19		55		10		6
male	42	*28*	12	*40*	19	*21*	5	*26*	21	*38*	6	*60*	2
female	108	*72*	18	*60*	70	*79*	14	*74*	34	*62*	4	*40*	4
18-40 years	32	*21*	5	*17*	20	*22*	4	*21*	11	*20*	1	*10*	1
40-60 years	74	*49*	16	*53*	47	*53*	11	*58*	24	*44*	5	*50*	3
>60 years	44	*29*	9	*30*	22	*25*	4	*21*	20	*36*	4	*40*	2
KS	15	*10*	6	*20*	8	*9*	5	*26*	5	*9*	0	*0*	2
cervical cancer	75	*50*	13	*43*	60	*67*	10	*53*	13	*24*	3	*30*	2
oesophageal cancer	46	*31*	5	*17*	19	*21*	3	*16*	25	*45*	2	*20*	2
liver cancer	14	*9*	6	*20*	2	*2*	1	*5*	12	*22*	5	*50*	
married	93	*62*	16	*53*	52	*58*	8	*42*	37	*67*	7	*70*	4
single/separate/divorce	21	*14*	7	*23*	14	*16*	7	*37*	7	*13*	0	*0*	
widowed	36	*24*	7	*23*	23	*26*	4	*21*	11	*20*	3	*30*	2
carers	121		25		64		15		54		9		3
male	34	*23*	5	*20*	17	*27*	2	*13*	17	*31*	3	*33*	
female	87	*58*	20	*80*	47	*73*	13	*87*	37	*69*	6	*67*	3
18-39	59	*39*	10	*40*	32	*36*	6	*32*	24	*44*	3	*30*	3
40-59	45	*30*	11	*44*	25	*28*	8	*42*	20	*36*	3	*30*	
60-89	17	*11*	4	*16*	7	*8*	1	*5*	10	*18*	3	*30*	

Kaplan Meier survival estimates

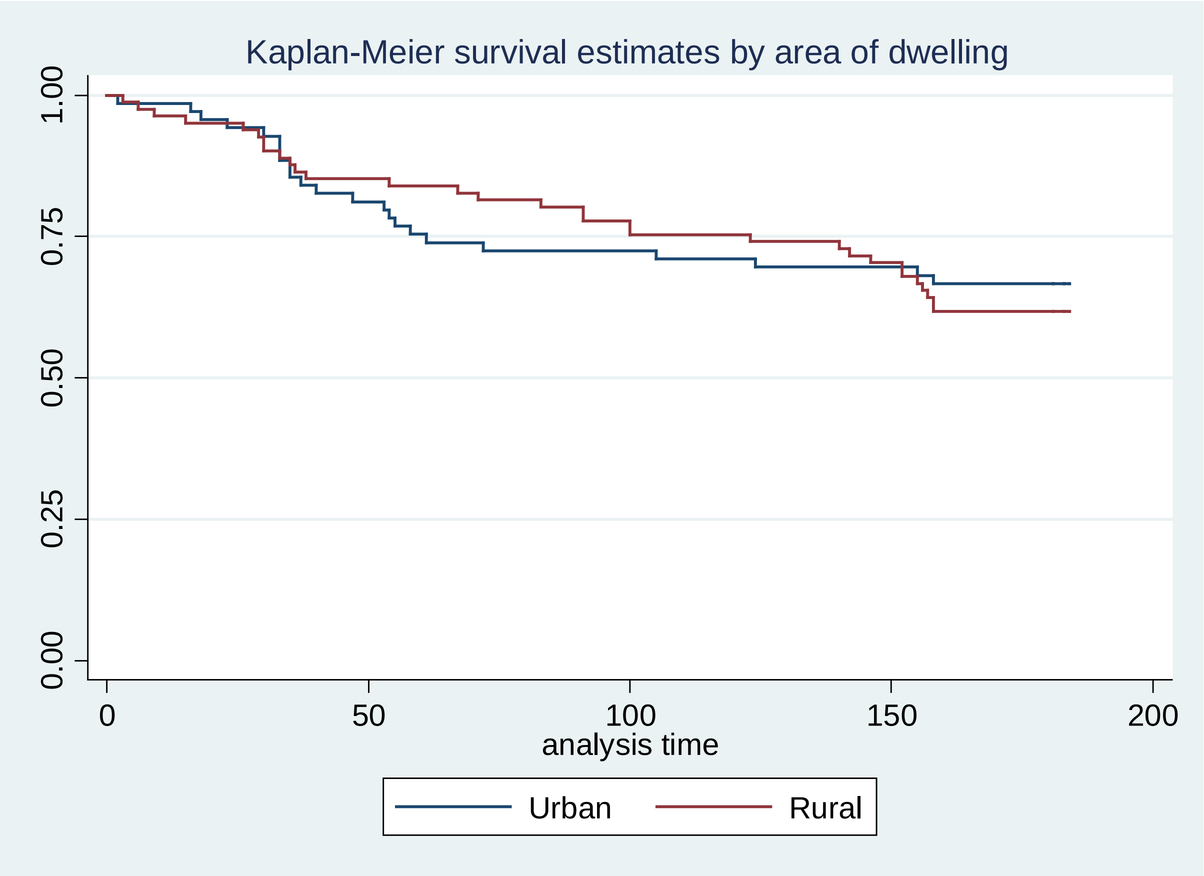

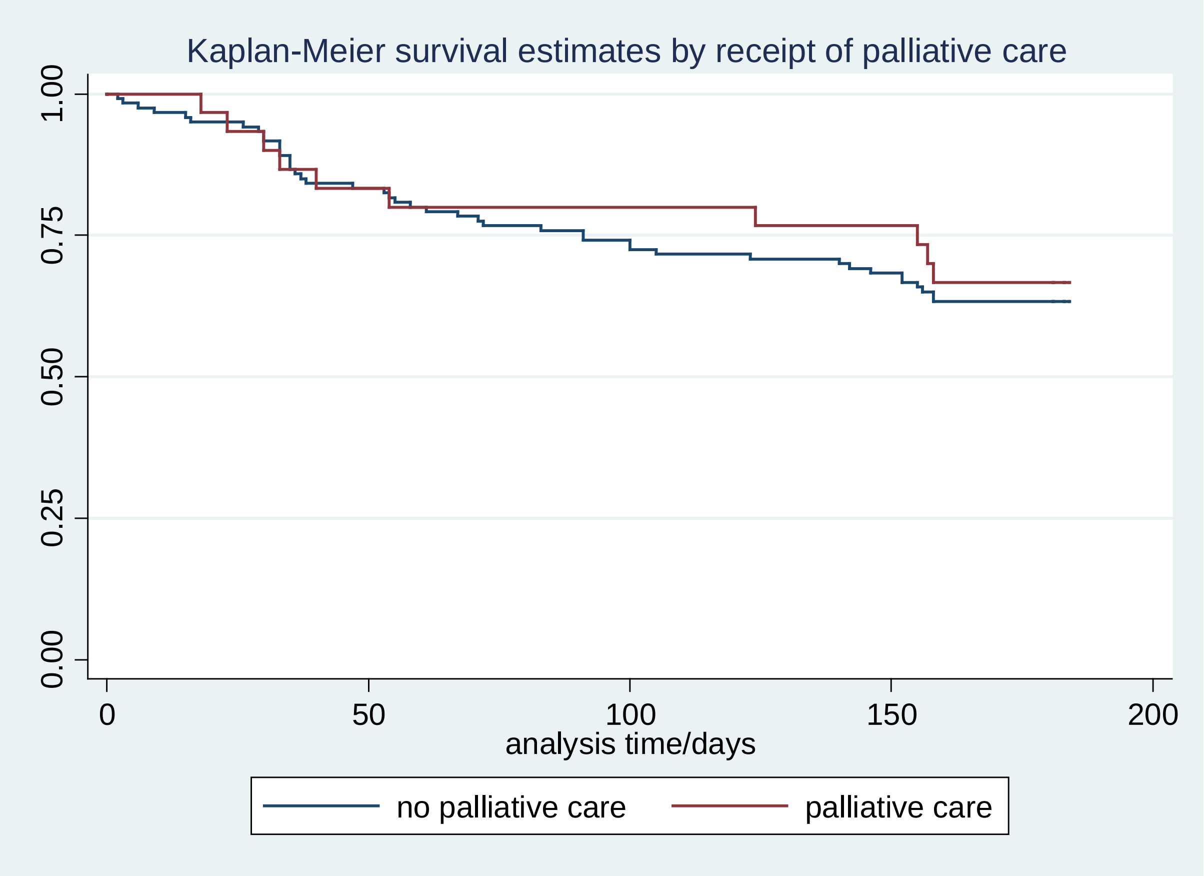

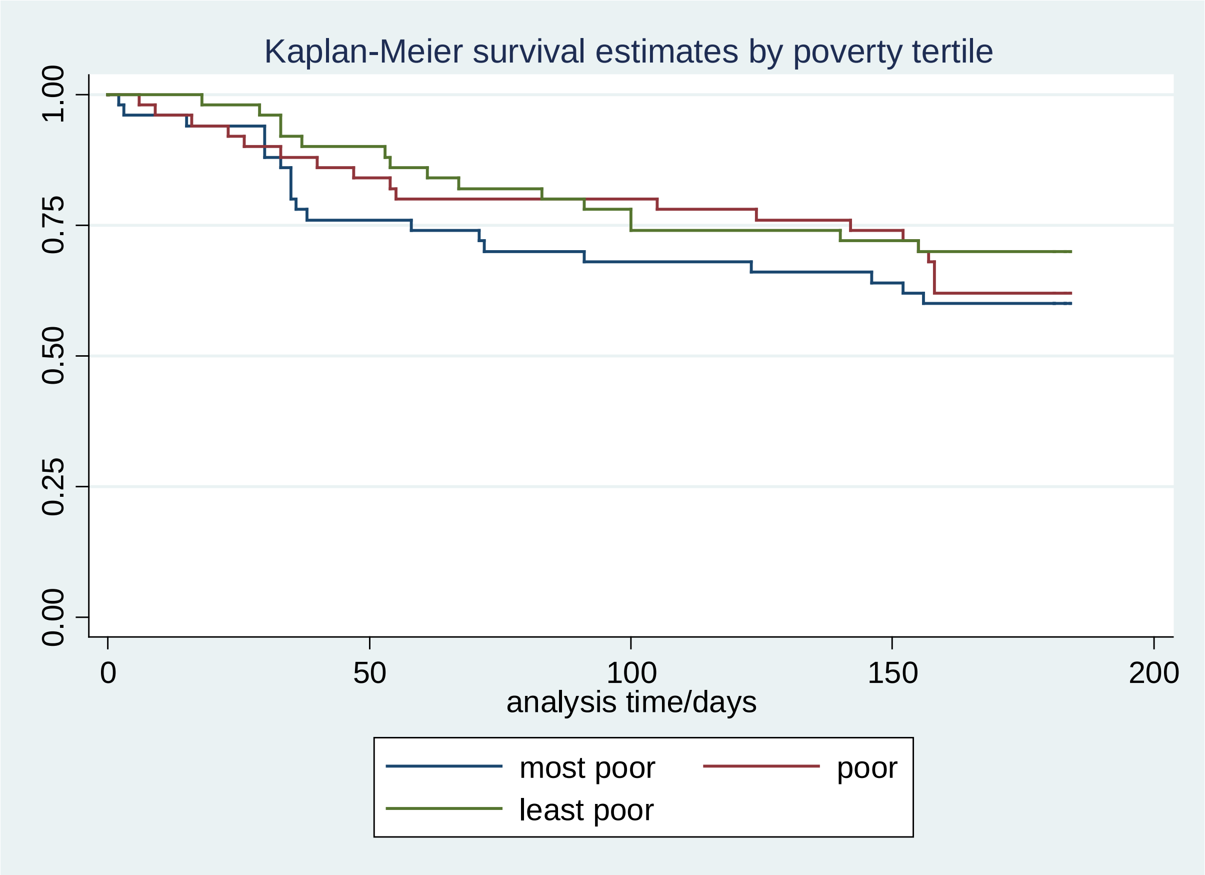
Kaplan-Meier survival estimates by poverty tertile
1.00
0.75
0.50
0.25
0.00
0
50
100
150
200
analysis time/days
most poor
poor
least poor

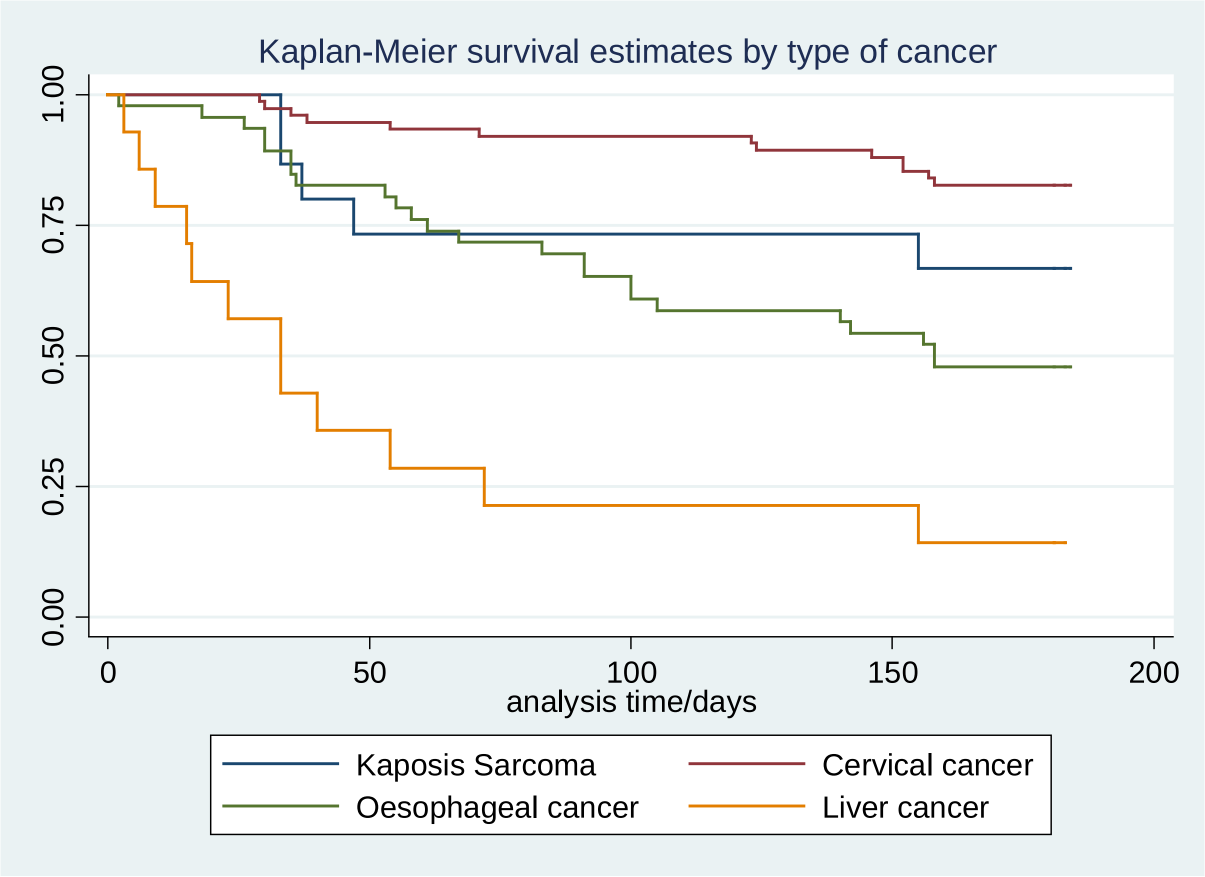
Kaplan-Meier survival estimates by type of cancer
1.00
0.75
0.50
0.25
0.00
0
50
100
150
200
analysis time/days
Kaposis Sarcoma
Cervical cancer
Oesophageal cancer
Liver cancer

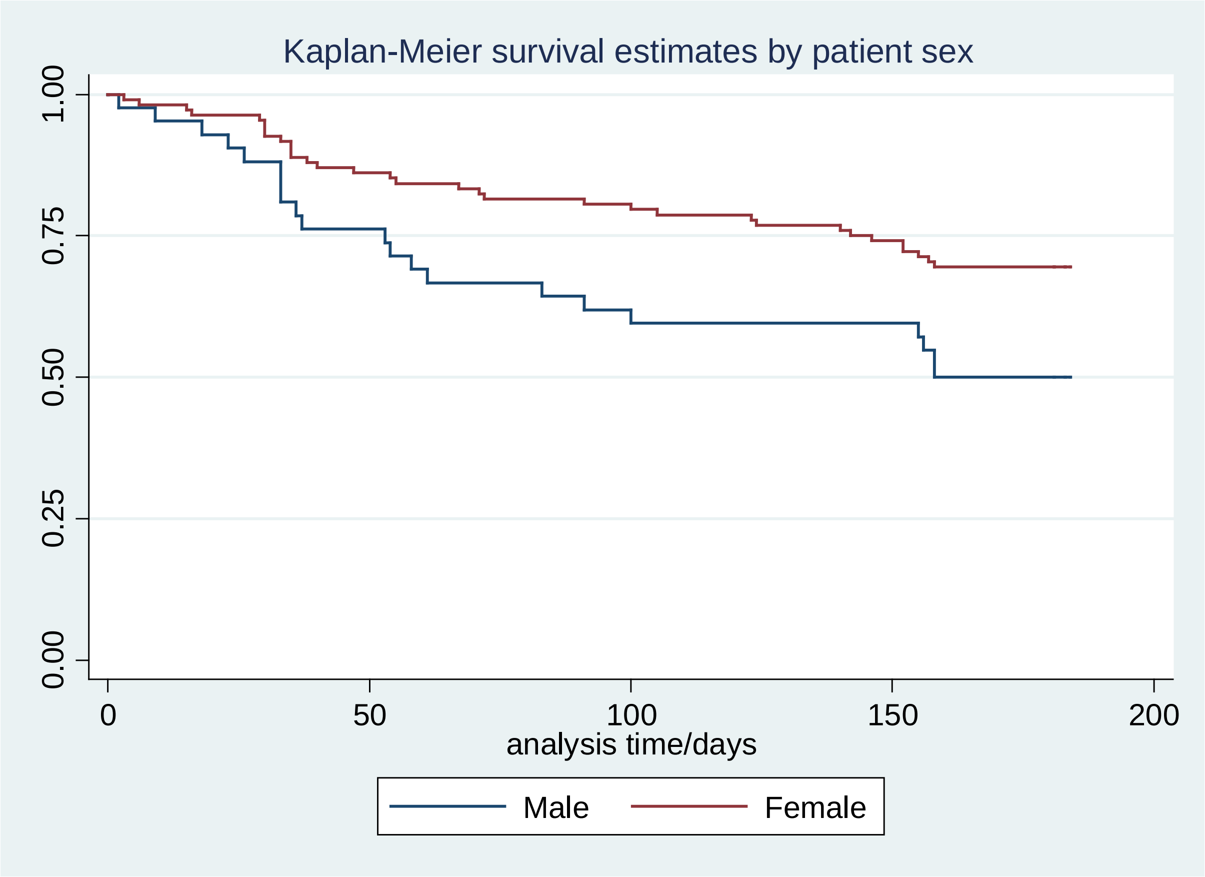

Kaplan-Meier survival estimates by patient sex
1.00
0.75
0.50
0.25
0.00
0
50
100
150
200
analysis time/days
Male
Female

Health related quality of life (HRQoL) utility scores for patients and carers at baseline and six months by receipt of palliative care (with bootstrapped mean and 95% CI)

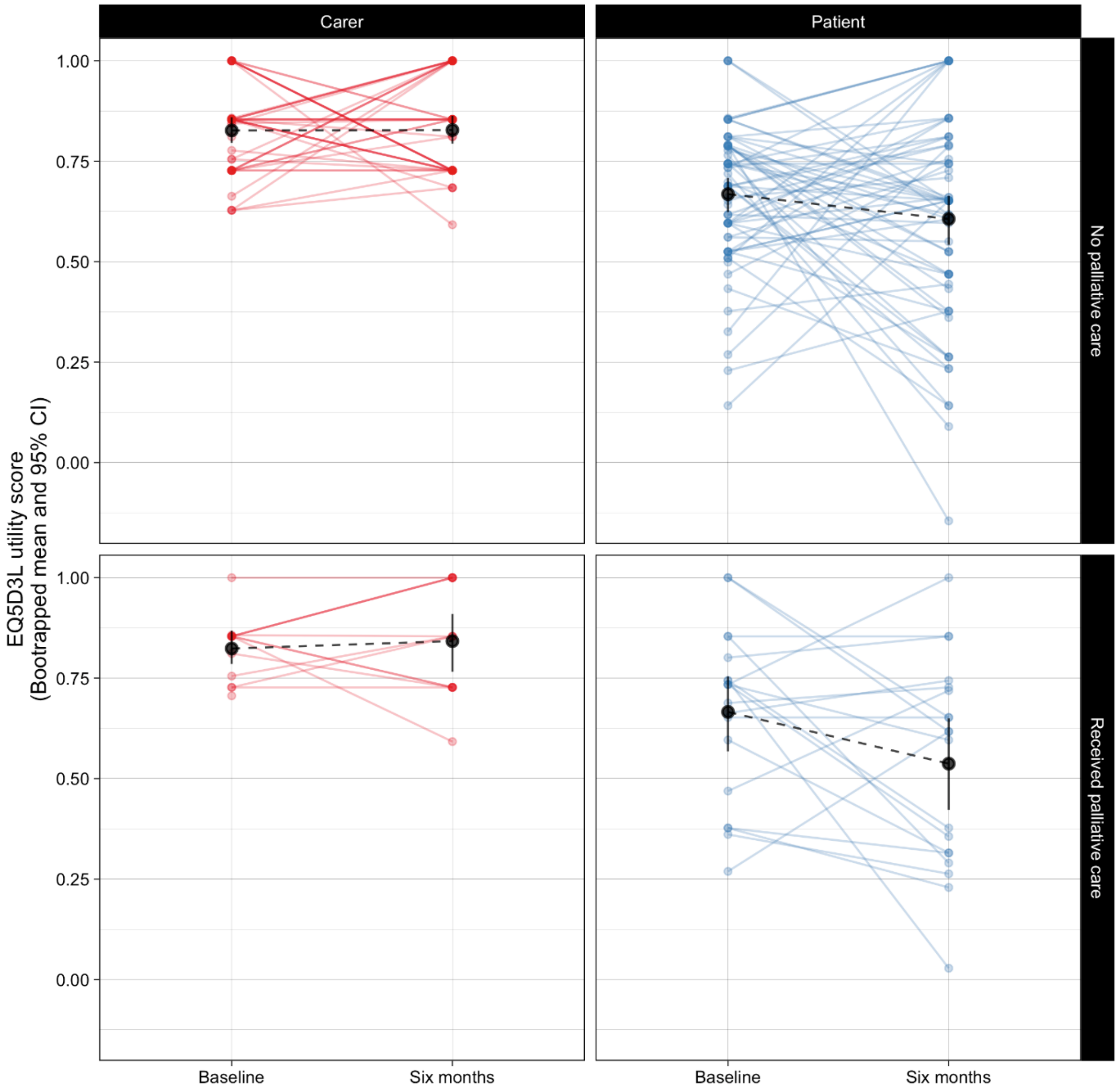

Proxy Means Test for Poverty (urban)

Questions

1) Please think about the person who is the head of your household. It may be you, or it may be someone else. What is the highest educational qualification the household head has acquired?

NONE == 1 (ref: 0)
PSLC == 2 (-0.1070230)
JCE == 3 (-0.4727860)
MSCE == 4 (-0.3210555)
NON-UNIVERSITY DIPLOMA == 5 (-14.5500879)
UNIVERSITY DIPLOMA/DEGREE == 6 (-14.1740569)
POSTGRADUATE DEGREE == 7 (-13.8194385)

2) What does the head of your household sleep on? Please select one of the following:

BED & MATTRESS == 1 (ref: 0)
BED & MAT (GRASS) == 2 (0.6709013)
BED ALONE == 3 (0.3235319)
MATTRESS ON FLOOR == 4 (0.4599334)
MATT (GRASS) ON FLOOR == 5 (0.7354924)
CLOTH/SACK ON FLOOR == 6 (2.9858984)
FLOOR (NOTHING ELSE) == 7 (0.0000000)
OTHER == 8 (-0.6208952)

3) How many people live in your household? Please count all the people who normally live with you and eat meals together. Include yourself when counting.
INTEGER RESPONSE (0.6222069)

4) Do you have working electricity in your dwelling?

YES == 1 (ref: 0)
NO == 2 (0.9590447)

5) Do you, either by yourself or together with another household member or someone outside your household, currently have an account at a bank, credit union, micro finance institution, village savings organization, or another financial institution?

YES ==1 (ref: 0) -
NO == 2 (0.8831791) -

6) In the past 7 days, did you worry that your household would not have enough food?

YES == 1 (ref: 0) -
NO == 2 (-0.5573320) -

7) Concerning your household's clothing, which of the following is true?

It was less than adequate for household needs == 1 (ref: 0)
It was just adequate for household needs == 2 (-0.6375926)
It was more than adequate for household needs == 3 (-0.9265472)

8) Does your household own a tape or CD/DVD player or a HiFi?
YES == 1 (ref: 0)
NO == 2 (0.8729641)

9) Does your household own an upholstered chair (armchair) or sofa set?

YES == 1 (ref: 0) - NO == 2 (0.9942764)

10) Does your household own an iron for pressing clothes?

YES == 1 (ref: 0)
NO == 2 (1.3393567) –

11) Imagine six steps, where on the bottom, the first step, stand the poorest people, and on the highest step, the sixth, stand the rich. SHOW THE PICTURE OF THE STEPS. On which step are you today?

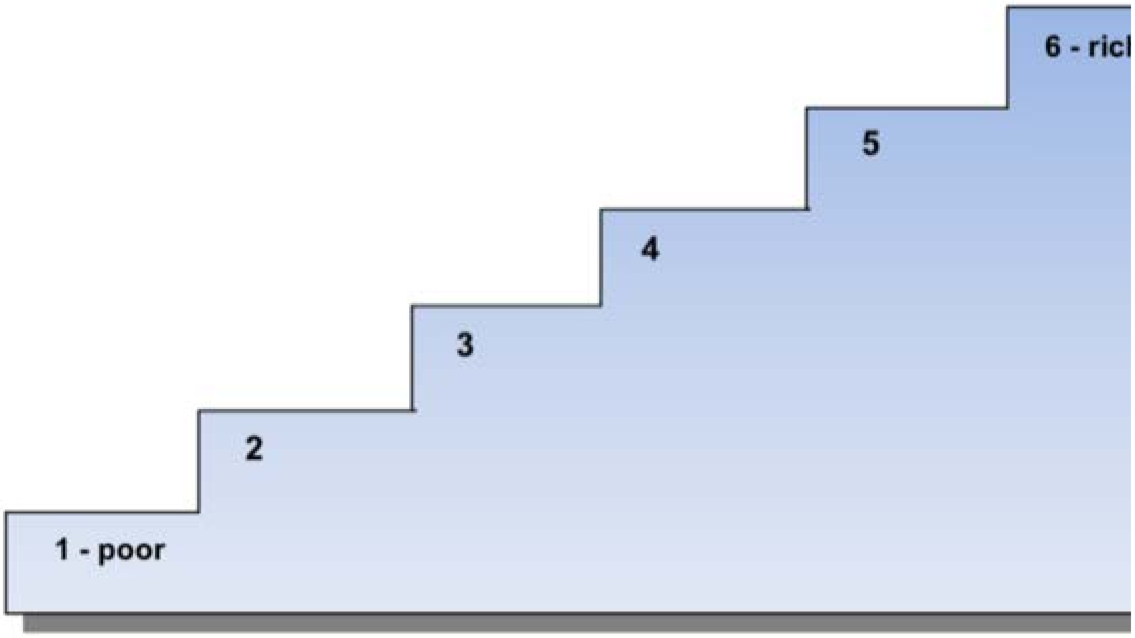

STEP 1 == 1
STEP 2 == 2
STEP 3 == 3
STEP 4 == 4
STEP 5 == 5
STEP 6 == 6

Deviations from the published study protocol

The following analyses were planned but not undertaken:

a) Poisson regression : details of the number of palliative care visits could not be reliably confirmed from available data sources (patient files, hospital records)

b) Cox proportional hazard models were not done. Log rank testing reported no unexpected significant differences in survival. Only where disaggregated by cancer type and patient sex (Patient sex was closely to cancer type in this study).

c) Changes in symptom burden using the Integrated Palliative Care Outcome Scale : this tool requires further validation in the local setting

Checklists

S1 STROBE checklist

**Section/Topic**	Item #	Recommendation	Reported on page/line #
**Title and abstract**	1	(*a*) Indicate the study's design with a commonly used term in the title or the abstract	1/1
		(*b*) Provide in the abstract an informative and balanced summary of what was done and what was found	2 and 3
Introduction			
Background/rationale	2	Explain the scientific background and rationale for the investigation being reported	4/14-24 (and previously published protocol)
Objectives	3	State specific objectives, including any prespecified hypotheses	5/10
Methods			
Study design	4	Present key elements of study design early in the paper	5/17
Setting	5	Describe the setting, locations, and relevant dates, including periods of recruitment, exposure, follow-up, and data collection	6/1-<br>7/9
Participants	6	(*a*) Give the eligibility criteria, and the sources and methods of selection of participants. Describe methods of follow-up	6/23-7/14 Appendix section 2
		(*b*) For matched studies, give matching criteria and number of exposed and unexposed	n/a
Variables	7	Clearly define all outcomes, exposures, predictors, potential confounders, and effect modifiers. Give diagnostic criteria, if applicable	Appendix section 1 Appendix section 2
Data sources/ measurement	8*	For each variable of interest, give sources of data and details of methods of assessment (measurement). Describe comparability of assessment methods if there is more than one group	7/ 5-15 Appendix section 1
Bias	9	Describe any efforts to address potential sources of bias	7/4, 7/14-15,
Study size	10	Explain how the study size was arrived at	8/5-11
Quantitative variables	11	Explain how quantitative variables were handled in the analyses. If applicable, describe which groupings were chosen and why	Appendix section 1
Statistical methods	12	(*a*) Describe all statistical methods, including those used to control for confounding	8/5-7
		(*b*) Describe any methods used to examine subgroups and interactions	8/4- 9/12
		(*c*) Explain how missing data were addressed	10/3-5
		(*d*) If applicable, explain how loss to follow-up was addressed	n/a
		(*e*) Describe any sensitivity analyses	Appendix section 1 and section 3
Results			
Participants	13*	(a) Report numbers of individuals at each stage of study—eg numbers potentially eligible, examined for eligibility, confirmed	Fig 1 Appendix section 4

**Section/Topic**	Item #	Recommendation	Reported on page/line #
		eligible, included in the study, completing follow-up, and analysed	
		(b) Give reasons for non-participation at each stage	Fig 1
		(c) Consider use of a flow diagram	Fig 1
Descriptive data	14*	(a) Give characteristics of study participants (eg demographic, clinical, social) and information on exposures and potential confounders	Table 1 and appendix section 4
		(b) Indicate number of participants with missing data for each variable of interest	appendix section 4
		(c) Summarise follow-up time (eg, average and total amount)	Fig 1 Appendix section 5
Outcome data	15*	Report numbers of outcome events or summary measures over time	11/22 Appendix section 4
Main results	16	(*a*) Give unadjusted estimates and, if applicable, confounder-adjusted estimates and their precision (eg, 95% confidence interval). Make clear which confounders were adjusted for and why they were included	Table 2
		(*b*) Report category boundaries when continuous variables were categorized	Table 2
		(*c*) If relevant, consider translating estimates of relative risk into absolute risk for a meaningful time period	Table 3
Other analyses	17	Report other analyses done—eg analyses of subgroups and interactions, and sensitivity analyses	Appendix section 1 and 3
Discussion			
Key results	18	Summarise key results with reference to study objectives	14/2-12
Limitations	19	Discuss limitations of the study, taking into account sources of potential bias or imprecision. Discuss both direction and magnitude of any potential bias	17/22 – 18/19
Interpretation	20	Give a cautious overall interpretation of results considering objectives, limitations, multiplicity of analyses, results from similar studies, and other relevant evidence	14/1-3
Generalisability	21	Discuss the generalisability (external validity) of the study results	17/24-18/3
Other information			
Funding	22	Give the source of funding and the role of the funders for the present study and, if applicable, for the original study on which the present article is based	Done

STROBE Statement
Strengthening the reporting of observational studies in epidemiology

STROBE Statement—Items to be included when reporting observational studies in a conference abstract STROBE_checklist_conference_abstract_DRAFT_v03 1

Item	Recommendation	
Title	Indicate the study's design with a commonly used term in the title (e.g cohort, casecontrol, cross sectional)	1/1
Authors	Contact details for the corresponding author	1/32
Study design	Description of the study design (e.g cohort, case-control, cross sectional)	2/12
Objective	Specific objectives or hypothesis	2/9
Methods		
Setting	Description of setting, follow-up dates or dates at which the outcome events occurred or at which the outcomes were present, as well as any points or ranges on other time scales for the outcomes (e.g., prevalence at age 18, 1998-2007).	2/11-13
Participants		
	Cohort study—Give the most important eligibility criteria, and the most important sources and methods of selection of participants. Describe briefly the methods of follow-up<br>Case-control study—Give the major eligibility criteria, and the major sources and methods of case ascertainment and control selection<br>Cross-sectional study—Give the eligibility criteria, and the major sources and methods of selection of participants<br>Cohort study—For matched studies, give matching and number of exposed and unexposed<br>Case-control study—For matched studies, give matching criteria and the number of controls per case	2/13-153
Variables	Clearly define primary outcome for this report.	2/15-16
Statistical methods	Describe statistical methods, including those used to control for confounding	2/15
Results		
Participants	Report Number of participants at the beginning and end of the study	2/21
Main results	Report estimates of associations. If relevant, consider translating estimates of relative risk into absolute risk for a meaningful time period	2/24-30
	Report appropriate measures of variability and uncertainty (e.g., odds ratios with confidence intervals	2/26,28,30
Conclusions	General interpretation of study results	3/1-4

CHEERS Checklist

Section/topic	#	Recommendation	Reported on page #/ line #
**TITLE AND ABSTRACT**			
Title	1	Identify the study as an economic evaluation or use more specific terms such as "cost-effectiveness analysis", and describe the interventions compared.	1/1
Abstract	2	Provide a structured summary of objectives, perspective, setting, methods (including study design and inputs), results (including base case and uncertainty analyses), and conclusions.	2/line 9, 15,25,<br>3/line 1-4
Abstract			
**INTRODUCTION**			
Background and objectives	3	Provide an explicit statement of the broader context for the study.<br>Present the study question and its relevance for health policy or practice decisions.	4/3-8,<br>4/21<br>5/13
**METHODS**			
Target population and subgroups	4	Describe characteristics of the base case population and subgroups analysed, including why they were chosen.	6/23-7/5
Setting and location	5	State relevant aspects of the system(s) in which the decision(s) need(s) to be made.	6/1-14
Study perspective	6	Describe the perspective of the study and relate this to the costs being evaluated.	7/7-12
Comparators	7	Describe the interventions or strategies being compared and state why they were chosen.	n/a
Time horizon	8	State the time horizon(s) over which costs and consequences are being evaluated and say why appropriate.	5/19-22
Discount rate	9	Report the choice of discount rate(s) used for costs and outc1-4omes and say why appropriate.	n/a
Choice of health outcomes	10	Describe what outcomes were used as the measure(s) of benefit in the evaluation and their relevance for the type of analysis performed.	8/5, 9/15-23
Measurement of effectiveness	11a	*Single study-based estimates*: Describe fully the design features of the single effectiveness study and why the single study was a sufficient source of clinical effectiveness data	n/a
	11b	*Synthesis-based estimates*: Describe fully the methods used for identification of included studies and synthesis of clinical effectiveness data.	n/a
Measurement and valuation of preference based outcomes	12	If applicable, describe the population and methods used to elicit preferences for outcomes	n/a-

Section/topic	#	Checklist item	Reported on page #
Estimating resources and costs	13a	*Single study-based economic evaluation*: Describe approaches used to estimate resource use associated with the alternative interventions. Describe primary or secondary research methods for valuing each resource item in terms of its unit cost. Describe any adjustments made to approximate to opportunity costs.	n/a
	13b	*Model-based economic evaluation*: Describe approaches and data sources used to estimate resource use associated with model health states. Describe primary or secondary research methods for valuing each resource item in terms of its unit cost. Describe any adjustments made to approximate to opportunity costs.	n/a
Currency, price date, and conversion	14	Report the dates of the estimated resource quantities and unit costs. Describe methods for adjusting estimated unit costs to the year of reported costs if necessary. Describe methods for converting costs into a common currency base and the exchange rate.	10/13<br>Appendix section 1
Choice of model	15	Describe and give reasons for the specific type of decision-analytical model used. Providing a figure to show model structure is strongly recommended.	n/a
Assumptions	16	Describe all structural or other assumptions underpinning the decision-analytical model.	n/a
Analytical models	17	Describe all analytical methods supporting the evaluation. This could include methods for dealing with skewed, missing, or censored data; extrapolation methods; methods for pooling data; approaches to validate or make adjustments (such as half cycle corrections) to a model; and methods for handling population heterogeneity and uncertainty.	9/5-13<br>Appendix section 1
**RESULTS**			
Study parameters	18	Report the values, ranges, references, and, if used, probability distributions for all parameters/ Report reasons or sources for distributions used to represent uncertainty where appropriate. Providing a table to show the input values is strongly recommended.	Tables 1, 4,5<br>Appendix section 4
Incremental costs and outcomes	19	For each intervention, report mean values for the main categories of estimated costs and outcomes of interest, as well as mean differences between the comparator groups. If applicable, report incremental cost-effectiveness ratios.	12/7-18
Characterising uncertainty	20a	*Single study-based economic evaluation*: Describe the effects of sampling uncertainty for the estimated incremental cost and incremental effectiveness parameters, together with the impact of methodological assumptions (such as discount rate, study perspective).	n/a
	20b	*Model-based economic evaluation*: Describe the effects on the results of uncertainty for all input parameters, and uncertainty related to the structure of the model and assumptions.	n/a

Characterising heterogeneity	21	If applicable, report differences in costs, outcomes, or cost-effectiveness that can be explained by variations between subgroups of patients with different baseline characteristics or other observed variability in effects that are not reducible by more information.	Table 2 Appendix section 4
**Section/topic**	**#**	**Checklist item**	
**DISCUSSION**			
Study findings, limitations, generalisability, and current knowledge	22	Summarise key study findings and describe how they support the conclusions reached. Discuss limitations and the generalisability of the findings and how the findings fit with current knowledge.	14/1-12, 18/3-11
**Other**			
Source of funding	23	Describe how the study was funded and the role of the funder in the identification, design, conduct, and reporting of the analysis. Describe other non-monetary sources of support.	Done
Conflicts of interest	24	Describe any potential for conflict of interest of study contributors in accordance with journal policy. Int eh absence of a journal policy, we recommend authors comply with International Committee of Medical Journal Editors recommendations.	Done
